# Smartphone-based Structure-from-Motion for the remote assessment of trunk rotation in spine deformity

**DOI:** 10.1016/j.xnsj.2026.100900

**Published:** 2026-05-17

**Authors:** Sinduja Suresh, Addison Elise Suhr, Penelope De Gavelle De Roany, Leanne Kok, Ella Morsch, Annabelle Stubbs, Maree Therese Izatt, Robert David Labrom, Geoffrey Noel Askin, Judith Paige Little

**Affiliations:** a Biomechanics and Spine Research Group (BSRG) at the Centre for Children’s Health Research (CCHR), School of Mechanical Medical and Process Engineering, Faculty of Engineering, Queensland University of Technology, 62 Graham St, South Brisbane, Queensland 4101, Australia; b Centre for Biomedical Technologies (CBT), Queensland University of Technology, 60 Musk Ave, Kelvin Grove, Brisbane, Queensland 4059, Australia; c Faculty of Engineering, School of Mechanical, Medical and Process Engineering, Queensland University of Technology, 2 George St, Brisbane City (CBD), Brisbane, Queensland 4000, Australia; d Orthopaedics Department, Queensland Children’s Hospital (QCH), 501 Stanley St, South Brisbane, Queensland 4101, Australia

**Keywords:** Adolescent idiopathic scoliosis, Telehealth, Surface topography, Structure from motion, Axial trunk rotation, Spine deformity

## Abstract

**Background:**

In scoliosis care, telehealth typically focuses on virtual assessments of posture and flexibility as surrogates for physical examinations, with quantitative measurements limited to radiography. This study evaluates the feasibility of using smartphone-based structure-from-motion (SfM) for patients to capture reliable 3D representations of their torsos using accessible, low-cost tools and enable remote surface measurement of Axial Trunk Rotation (ATR) without physical scoliometer use. We assess the accuracy and reliability of ATR measurements derived from SfM models, identify recommended image capture conditions, and examine the influence of reconstruction parameters such as tie point number, depth map quality, manual background masking, and scaling.

**Methods:**

Twenty adolescents with idiopathic scoliosis were recruited from a single hospital and 3D surface scanning (3DSS) and SfM datasets were acquired during standard appointments. A structured protocol for image capture and reconstruction was developed. ATR measurements from SfM were compared with clinical (analog scoliometer) and technical (3DSS) gold standards using correlation and Bland-Altman plots. ATR from SfM models scaled in 2 software packages was compared with 3DSS. Intra and inter-user variability in manual image masking was assessed on a subset of 5 patients captured under varying background conditions.

**Results:**

A total of 60 to 80 photographs captured in a structured manner provided the best balance between time, patient fatigue, and reconstruction quality. Optimal reconstruction occurred with 15,000 tie points and medium-quality depth maps. ATR measured on SfM models showed strong positive correlation (>0.85, p < .001) with both the analog scoliometer and 3DSS, with proportional biases of 1.13° and 1.33° (≥90% points within agreement limits). Scaling comparisons showed mean biases of 0.78° (Agisoft Metashape) and 1.13° (Geomagic Wrap). Manual masking produced ATR variability consistently <2°.

**Conclusions:**

Smartphone-based SfM offers a practical, low-cost alternative to 3DSS for evaluating trunk rotation. Its accuracy and reliability support its potential integration into telehealth workflows for scoliosis care.

## Introduction

Adolescent Idiopathic Scoliosis (AIS) is a three-dimensional spinal deformity characterized by abnormal lateral curvature of the spine with vertebral and ribcage rotation [1]. Its prevalence can vary depending on geographical region, sex (tenfold higher prevalence in females), age, skeletal maturity, and family history, but it is most cited in the literature as being between 3% and 5% of the general population [[2], [3], [4]]. The condition typically progresses during growth and, if left untreated, can cause visible torso asymmetry, cardiopulmonary compromise, and pain.

The majority of abnormal spine curvatures in children are first detected by individuals in the community with no medical training [5] before they are seen in primary care. In Australia, primary care is the first contact a patient has with the healthcare system, and this could be with a medical practitioner, pharmacist or an allied health professional such as a physiotherapist [6]. A recent analysis of the ScoliCare database in Australia shows that children with AIS presenting to primary care tend to already be at “moderate severity” (median scoliosis curve (Cobb) angle of 26°) [7]—the stage at which conservative treatment with rigid orthoses (spinal braces) would typically be prescribed at specialist tertiary care clinics. The time taken for a patient to make their way from primary to tertiary care is significant, with one study in Western Australia estimating an average 20.5 months from symptom detection to specialist review [8]. As a result, many patients arrive at tertiary care clinics too late for conservative treatment to be effective, with the average Cobb angle at first specialist review reported to be as high as 49.5° [8] (well beyond the recommended bracing threshold of 25° to 40° [9]). A similar trend has been observed in other countries as well [5,[10], [11], [12]] with concerns reported around reduced conservative treatment options, increased surgery rates, and cost.

Geographical inequity exacerbates these challenges. Approximately 27% of Australians live outside major cities [13], and those in regional and remote communities experience disproportionately higher rates of delayed diagnosis and avoidable hospitalization. The temporal, financial, logistical, and emotional burden associated with repeated travel for specialist assessment further compounds access barriers for families of children with scoliosis [14].

**Fig. 1:**
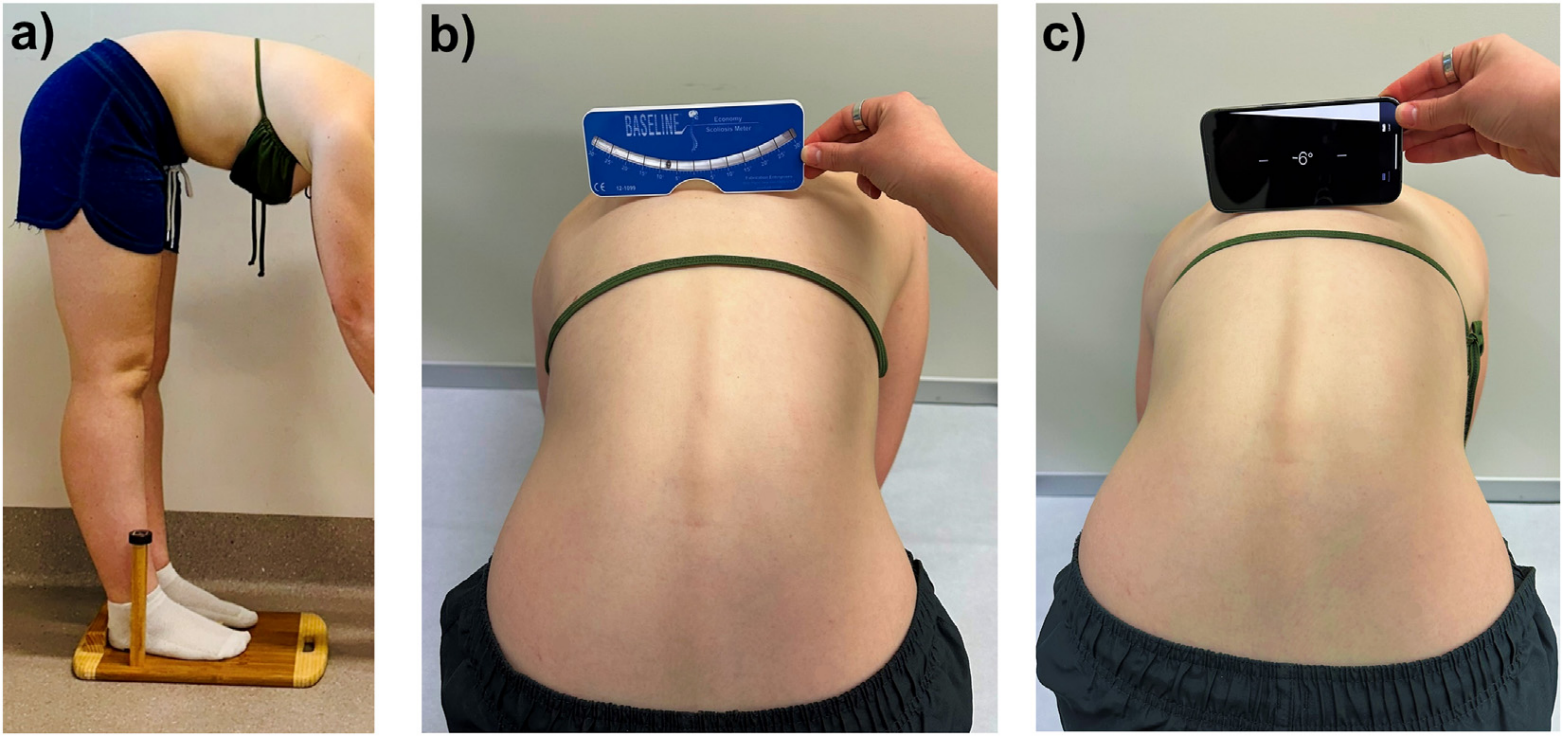
ATR measurement using a scoliometer. (A) The AFB position, (B) the ATR measured in this position with the analog scoliometer, (C) the ATR measured in this position with a smartphone inclinometer [19].

Telehealth offers a potential mechanism to mitigate these inequities by extending specialist services beyond urban boundaries. Although its value in improving access is well established [15,16], most current telehealth systems remain limited in their ability to replicate the quality and comprehensiveness of face-to-face assessment. The COVID-19 pandemic highlighted this gap [17,18], accelerating global interest in digital technologies capable of supporting remote musculoskeletal examination [14]. In scoliosis management, emerging telehealth strategies have focused on virtual assessments of posture, flexibility, and torso shape as surrogates for traditional physical examinations, but quantitative measurements remain limited to radiography. Axial Trunk Rotation (ATR) is typically measured in the clinic using an analog scoliometer with the patient in the Adam’s Forward Bend (AFB) position. Where the ATR is beyond the 30° measurement limit of the analog scoliometer, a smartphone inclinometer (physical digital proxy for analog scoliometer measurements) is sometimes used (Fig. 1). Accurate remote surface measurement of the ATR (virtual digital proxy for scoliometer measurements) would require patients to capture reliable 3D representations of their bodies using accessible, low-cost technology [19].

Photogrammetry, first used in geographical mapping and terrain visualizations in 1849 [20], gained popularity in the medical and anthropometry fields in the 1970s for being able to generate 3D surface topography of bony anatomy. The process requires professional photography equipment and generally relies on the object being motionless, lighting conditions to be consistent with no interference from shadows, and the background to be bland and static [21,22]. High-performance photogrammetry setups intended for humans (complex geometry, nonstationary) therefore require clutter-free spaces and typically allow for several photos to be captured from an array of cameras at a single instant to reduce artifacts from movement (such as breathing and postural sway), airflow or lighting changes [23]. Such systems are large, costly to install and maintain [24], cannot be easily moved once installed, and require trained personnel to operate, maintain, and interpret the results. As such, the widespread implementation of high-performance photogrammetry systems in the average medical clinic has been difficult, and their integration into telehealth pathways for home-based use has been impractical.

Advances in smartphone imaging quality and computational processing have renewed interest in low-cost alternatives to traditional photogrammetry equipment and methods [25]. Conventional photogrammetry relies on the use of marker points placed on the subject before capturing images. These markers serve as reference points for 3D reconstruction. While markers enhance accuracy in controlled environments, they are less effective in dynamic or uncontrolled settings such as a person’s home. Structure-from-motion (SfM), on the other hand, is a contemporary photogrammetry technique wherein 3D structures are derived from a sequence of 2D images, thus only being relevant to single-camera setups. The process involves identifying common features in overlapping images and establishing their spatial relationships [26]. SfM is versatile and widely used, and is particularly suited to smartphone use, due to its ability to reconstruct scenes without relying on predefined markers. While SfM has been successful in capturing stationary objects and humans (adults) in controlled environments [27,28], capturing suitable image data of humans in natural and uncontrolled environments remains challenging [29].

This study investigates the feasibility of smartphone-based SfM for use in telehealth for pediatric scoliosis. We investigate the accuracy and reliability of measuring the ATR, a clinically relevant surface metric that can be measured from 3D reconstructions of the patient’s torso. Additionally, we report on the impact of SfM reconstruction parameters such as manual background masking of photographs and 3D model scaling. This approach has the potential to improve equity of access, reduce travel burdens, and generate substantial cost savings, while maintaining clinical rigor in the ongoing management of pediatric scoliosis.

## Methods

3D models of AIS patients were created using 2 surface topography methods: 3D surface scanning (3DSS) and SfM. 3DSS was performed using the high-fidelity Artec Leo scanner (Artec3D, Luxembourg), and photographs for SfM were captured using a Samsung Galaxy S22 smartphone. Artec scanners are considered the gold standard in 3DSS technology, and 3D models obtained from them have been shown to be highly accurate and reliable [[30], [31], [32]]. Therefore, the 3DSS dataset was used as a control for comparison with the SfM counterpart.

### Dataset and demographics

A cohort of 20 AIS patients from the Queensland Children's Hospital Spine Outpatient Clinic, Brisbane, Australia was recruited for the study, from which datasets of both 3DSS control models and SfM models were generated during a standard medical appointment. Participants of both sexes (male = 3, female = 17), with a mean age of 14.78 ± 1.42 years, a mean body mass index of 20.35 ± 3.86 kg/m^3^ (range 15.3–23.31 kg/m^3^) were included. No Lenke scoliosis curve classification types [33] were specifically excluded (Type 1 = 15, Type 3 = 3, Type 5 = 2).

### Image capture protocol

#### 3DSS with handheld scanner

Each patient was scanned using the Artec Leo 3D scanner at 22 frames per second in the AFB pose, from which the ATR can be clinically measured. Details of patient positioning and scanning technique have also been described in previous publications [19,31]. In brief, patients were positioned on a wooden calibration board and provided a box support to minimize postural sway during the scan. Scanning was completed by one experienced operator (research team member), and the 360° scan path remained consistent for all patients.

#### Smartphone SfM

To enable accurate three-dimensional (3D) reconstruction using a single-camera photogrammetry approach, an image acquisition protocol was developed based on established recommendations from Agisoft Metashape (Agisoft LLC, St. Petersburg, Russia). They suggest capturing images at regular angular intervals (every 10°–15°) around the subject to achieve 70% to 80% overlap between consecutive images, with a total of 30 to 100 images distributed over 360° around the body. No other specific guidelines for human capture in special poses are established, so the exact number of images was determined as a trade-off between capture time, positioning fatigue, and obtaining sufficiently overlapping images for good quality 3D reconstruction.

Practical constraints associated with imaging children in the AFB pose were considered during protocol development. Based on over a decade of research experience acquiring 3DSS in a hospital setting, we find that children typically demonstrate limited tolerance for maintaining the AFB pose beyond 2 minutes. A trained researcher can acquire 80 to 100 images within this time when moving around the patient. However, a reduced minimum image set of 60 structured photos was defined to accommodate (1) variability in patient tolerance to prolonged AFB and (2) lay operators, such as parents or caregivers, who may require additional time to complete image acquisition, particularly during initial attempts. Where patient tolerance and operator speed permitted, an additional 20 images (for a total of 80) or more can be optionally captured to improve image overlap and reconstruction quality.

**Fig. 2:**
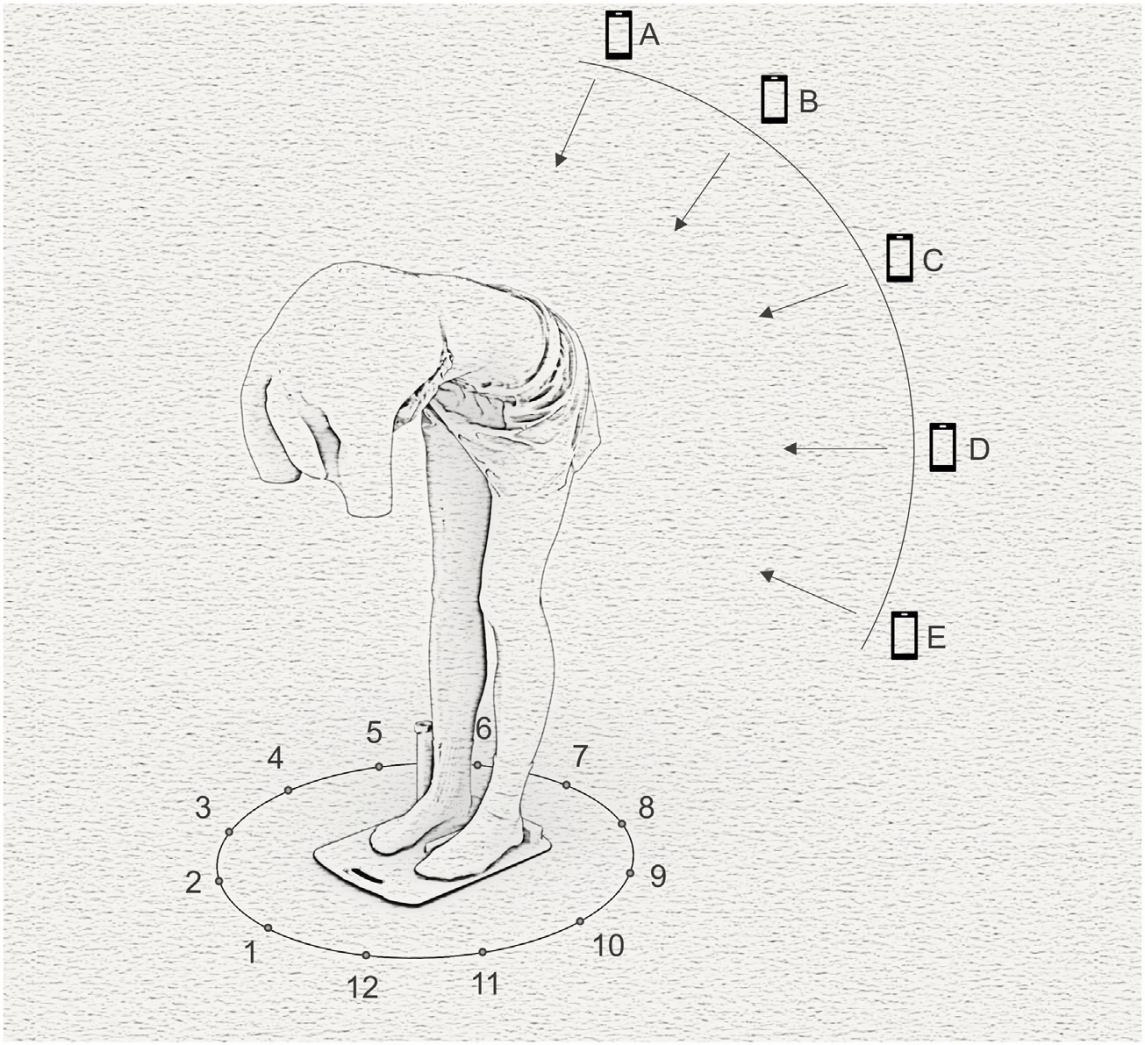
Schematic showing a child with AIS (deidentified) positioned in AFB. The photo capture protocol shows 12 positions (1–12) radially distributed around them. At each of these positions, 5 images were captured with a smartphone in an arc (A–E). Following this, 20 photos were captured around the patient at random for redundancy—this is not shown in the figure. Therefore, 80 photos minimum were captured in total.

Therefore, for this study, one experienced operator (research team member) captured photographs of each patient using a smartphone with a standard 12-megapixel camera. Images were taken from 12 positions radially distributed around the patient with 5 images taken in a vertical arc at each position (60 structured photos). This was followed by an additional subset of at least 20 photos taken at random positions around the patient (80 photos total minimum). Patients were positioned in the AFB pose on the same calibration board used for 3DSS. The patient positioning and structured photo capture protocol to obtain 60 photos are detailed in Fig. 2.

The SfM capture protocol developed as part of this study is unique, as photos were captured over approximately a 90-second period by hand (no standard positioning rig was used), of a subject who is breathing (causes breathing artifact), has some minor motion (due to natural postural sway when standing, and small arm movements due to fatigue), and with an uncontrolled background. While the background of the room was simple, it was impossible to remove all objects, such as standard clinic wall fixtures, tables, and chairs containing items required for the scanning appointment. In addition, at least one parent/guardian was often in close proximity, sitting on a chair surrounded by their belongings, and captured as a moving part of the otherwise stationary background.

### Raw data processing and 3D model generation

#### 3DSS with handheld scanner

The raw 3DSS captured using the Artec Leo scanner were imported into the associated software Artec Studio Professional (Version 17) to be stitched, refined, and fused into watertight 3D models using a standard operating procedure developed in-house. The resultant 3D models were exported in binary stereolithography (.stl) format for postprocessing.

#### Smartphone SfM

3D models for SfM were reconstructed using the Agisoft Metashape software. Existing reconstruction algorithms within the software were applied, along with custom background masking to create 3D models representing the patient’s body. The masking procedure involves hand-drawing a contour line around the subject of interest to separate it from the background. It is of high significance when attempting to obtain good quality 3D models taken under imperfect conditions (moving and/or cluttered background). Again, no clear guidelines exist for this, except that a mask must be drawn for an image whenever the background significantly changes (determined by visual inspection by the user), and that a small margin between the patient and the mask boundary must be applied when drawing the contour. With the photo capture configuration in this study, the background significantly changes every time the user takes a step around the patient (every 5 images). It was determined that 8 to 12 masks were most appropriate, with the exact number of masks defined by the user. This decision-making was dependent on patient-specific factors, such as the absence/presence of family members in the background or personal belongings, and the user’s interpretation of the severity of their movements across the captured images. For this application and photo capture protocol, the use of more masks than required did not result in an improved model quality.

After importing photos into Metashape, masks were drawn around the body of the patient to exclude the background and include the calibration board for scaling and alignment. Photos were then aligned by applying the masks to tie points, using a tie point limit of 15,000. Optimization of the tie point limit is shown in Supplementary Fig. 1 (qualitative data) and Supplementary Fig. 2 (quantitative data) for a 5-patient subset (chosen for assessing masking reliability described in subsequent sections). Metashape recommends starting with a tie point limit of 4,000 to 10,000 and only increasing the tie points to 20,000 and above if image alignment is difficult. Keeping the masking consistent, the goal was to achieve 100% alignment of images, tie point multiplicity of 3 to 6, and 100% generation of depth maps. In Supplementary Fig. 2, unacceptable outcomes are highlighted in red while acceptable outcomes are highlighted in green. Acceptable outcomes were consistently achieved at 15,000 tie points, and this value was then used for the full dataset of 20 patients. Increasing the tie point limit to 20,000 did not significantly improve the qualitative output or quantitative goals.

A 3D point cloud was generated from the masked tie points, which was further cropped to include the patient’s geometry only. A 3D surface mesh of each patient was then reconstructed from the 3D point cloud using medium-quality depth maps. Qualitative and quantitative optimization of depth map quality is shown in Supplementary Fig. 3. The main quantitative assessment parameter was model reconstruction time, while the main qualitative assessment parameters were feature detail, completeness of torso, and smoothness of the model.

Depth filtering was disabled due to the complex geometry of the human body and potential loss of useful data points. The resultant 3D models were exported in binary stereolithography (.stl) format for postprocessing.

#### 3D model postprocessing

Both the 3DSS and SfM scans were postprocessed (scaled, aligned, cropped, and smoothed) in Geomagic Wrap 2021 (Oqton, USA). Additionally, a second set of SfM models was prepared by employing the scaling operation within Metashape.

*Scaling:* Photogrammetry reconstructions require scaling as dimensional information cannot be triangulated from images. SfM models were scaled to the correct dimensions using the wooden calibration board (with known dimensions) on which the patients stood to be scanned. Two methods of scaling were compared in this study: (1) scaling natively within Agisoft Metashape, and (2) scaling the 3D model externally in Geomagic Wrap using the known dimensions of the wooden board. 3DSS models do not require scaling as this is automatically determined by the scanner.

*Alignment and cropping:* For this step, models from both 3DSS and photogrammetry methods were processed in the same way. The models were aligned to the world coordinate system using the wooden calibration board. The models were then cropped to only include the torso (head and limbs were removed), and any unwanted items captured in the background were deleted. In preparation for making digital measurements with the semi-automated tool (detailed in the next section), the anterior torso was cropped to a flat surface, leaving only the posterior torso from the shoulder to just below the left/right posterior superior iliac spines.

**Fig. 3:**
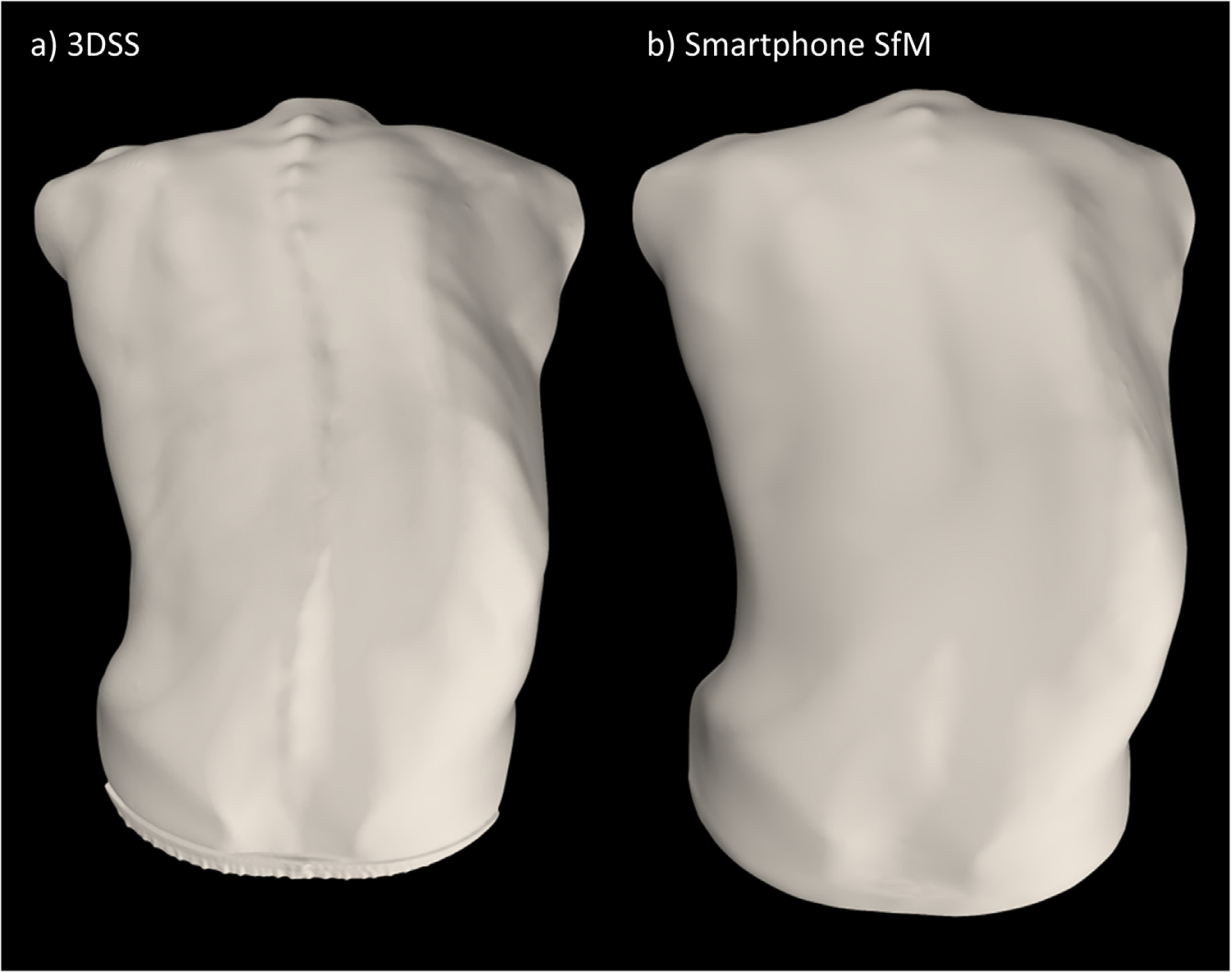
Fully processed deidentified 3D model for 2 capture types: (A) 3DSS and (B) Smartphone SfM.

*Smoothing:* Next, the models were globally smoothed. Excessive smoothing and noise reduction can result in loss of detail, so this operation was only performed once per model. The inbuilt “Mesh Doctor” tool was used to repair errors (such as nonmanifold edges, self-intersections, highly creased edges, spikes, small components, and small holes), if any, in the polygon surface mesh. High-resolution 3DSS meshes were then conservatively decimated to reduce the file size for further analysis. This reduction step was not required for the SfM models as they are already of small file size. An example of a fully processed and deidentified 3D model for each capture type is shown in Fig. 3.

### Measurement and analysis protocol

#### ATR measurements

The reconstruction outcomes of the 3DSS and SfM models were verified in a clinical context by comparing measurements of the ATR from each model. The ATR is a key clinical metric that is measured nonradiographically from the surface of a patient’s torso while the patient is in the AFB pose, to quantify the fixed vertebral rotational component of scoliosis. It is used throughout the scoliosis care trajectory (screening, diagnosis, treatment, monitoring) in conjunction with the radiographically measured spinal curve angles (Cobb angles).

The clinical ATR was recorded using the analog scoliometer at each patient scanning appointment by clinical staff. For 3DSS and SfM, the ATR was measured virtually on the reconstructed 3D model. Virtual digital measurements were facilitated by a semi-automated algorithm previously developed in-house on the Rhino7-Grasshopper (Robert McNeel and Associates, USA) software. This virtual digital scoliometer accurately mimics the functioning of the analog scoliometer on a computer, producing an ATR measurement when provided a 3D model of the patient. The algorithm has been previously validated for reliability, accuracy, sensitivity, and specificity [19].

#### ATR measurement comparison with technical and clinical gold standards

The Spearman correlation coefficient was calculated to obtain the relationship between the digital and analog scoliometer measurements. To further assess agreement in the values themselves, a Bland-Altman analysis was used to calculate measurement bias.

#### Influence of masking on ATR measurement

Masking reliability was evaluated by repeating background masking attempts and then following the established 3D model reconstruction pipeline to generate several 3D models on which the ATR was measured. This masking analysis was only performed for a maximum of 5 patients as the process is time-consuming (masking takes 10 min per patient, following which the images are aligned and then reconstructed, which takes a further 1.5 hours on average) and labor-intensive. The patients were chosen based on the condition of the room background and lighting on the day of image capture, with an aim to have the subset as varied as possible. Background conditions for each of the 5 patients is described in Supplementary Fig. 4.

Four users were asked to participate in the masking process. User A had some training and intermediate experience with the SfM reconstruction process. User B had significant experience with the SfM reconstruction process. Users C and D had no experience with SfM and were given brief instructions on how to do the masking on Metashape.

**Fig. 4:**
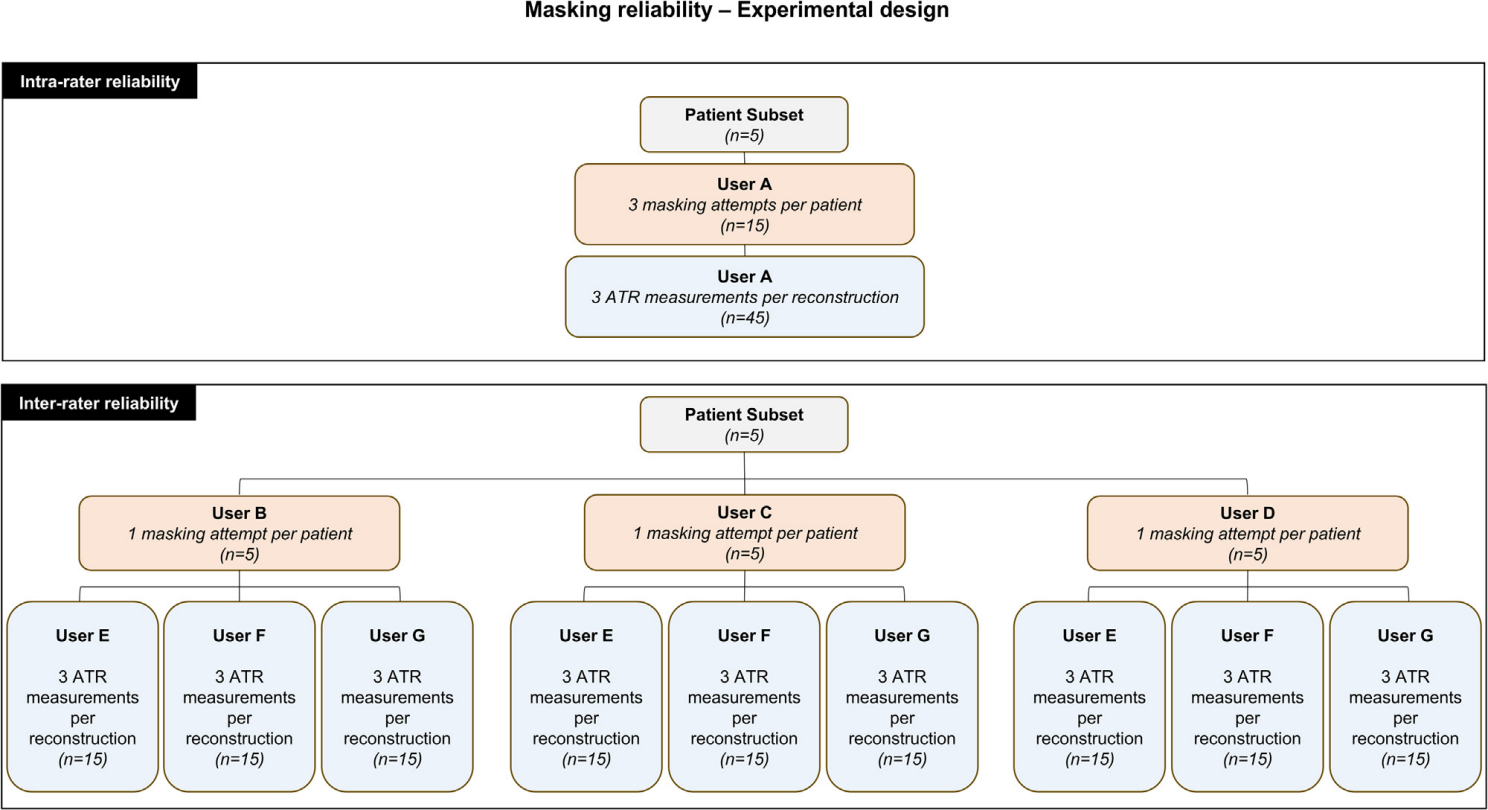
Schematic showing experimental procedure for assessing masking reliability.

User A repeated the masking procedure 3 times for 5 patients, reconstructed the 3D models using the optimized parameters, and then measured the ATR 3 times for each reconstruction. Users B, C, and D completed the masking procedure once on each of the 5 patient scans. Given the inexperience of Users C and D, the SfM reconstruction and ATR measurements for the inter-rater assessment were performed by 3 other Users E, F and G, who all had intermediate experience with SfM reconstruction and ATR measurement. Three digital ATR measurements were made for each reconstruction. A flowchart showing the experimental setup for this assessment is shown in Fig. 4. Mean absolute error for ATR measurements was compared between measurement attempts.

#### Influence of scaling method on ATR measurement

Bland-Altman plots were created to estimate the measurement bias in the ATR measurements when SfM models were scaled in 2 different software, Agisoft Metashape and Geomagic Wrap.

### Statistical analysis

Absolute measurement error was calculated on Microsoft Excel (Vsn 2510). Correlation plots and Bland-Altman analyses were performed on R statistical computing software (R Foundation for Statistical Computing, Vienna, Austria).

Spearman correlation coefficient was used to account for possible nonlinearity in the data. Significance levels were denoted as * (p < .05), ** (p < .01) and *** (p < .001).

Limits of Agreement (LOA) for the Bland-Altman analyses were calculated at a significance level of 0.95.

## Results

### ATR measurement comparison with technical and clinical gold standards

**Fig. 5:**
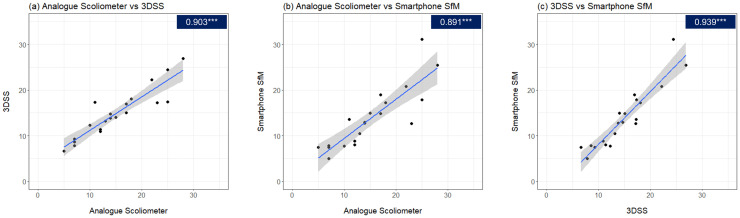
Scatterplots comparing ATR measurements in degrees for pairs of measurement methods (A) Analog Scoliometer versus 3DSS, (B) Analog Scoliometer versus Smartphone SfM, and (C) 3DSS versus Smartphone SfM. The linear line of best fit is indicated in blue, and the gray shading represents the standard error. The Spearman correlation coefficient for each pair is included in the top right corner in a blue box (***p < .001). For each plot, *n* = 20.

As denoted in Fig. 5, ATR measured on the SfM 3D models showed a high positive correlation (p < .001) with both the ATR measured using the analog scoliometer (clinical gold standard) and the ATR measured on the 3DSS models (technical gold standard).

**Fig. 6:**
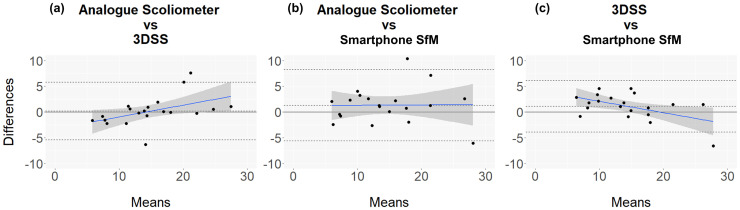
Bland-Altman plots for ATR measurements in degrees (A) Analog Scoliometer versus 3DSS, (B) Analog Scoliometer versus Smartphone SfM, and (C) 3DSS versus Smartphone SfM. The blue line represents the proportional bias, the gray shading represents the 95% confidence interval for the proportional bias line, and the dotted lines represent the limits of agreement. For each plot, *n* = 20.

Bland-Altman comparison of ATR (Fig. 6) between the analog scoliometer and 3DSS measurements reported an average proportional bias of 0.2°, with 90% of points within the 95% LOA. Comparison between the analog scoliometer and smartphone photogrammetry measurements reported an average bias of 1.13°, with 90% of points within the LOA. Comparison between 3DSS and smartphone photogrammetry measurements reported an average proportional bias of 1.33°, with 95% of points within the LOA.

### Influence of masking on ATR measurements

**Fig. 7:**
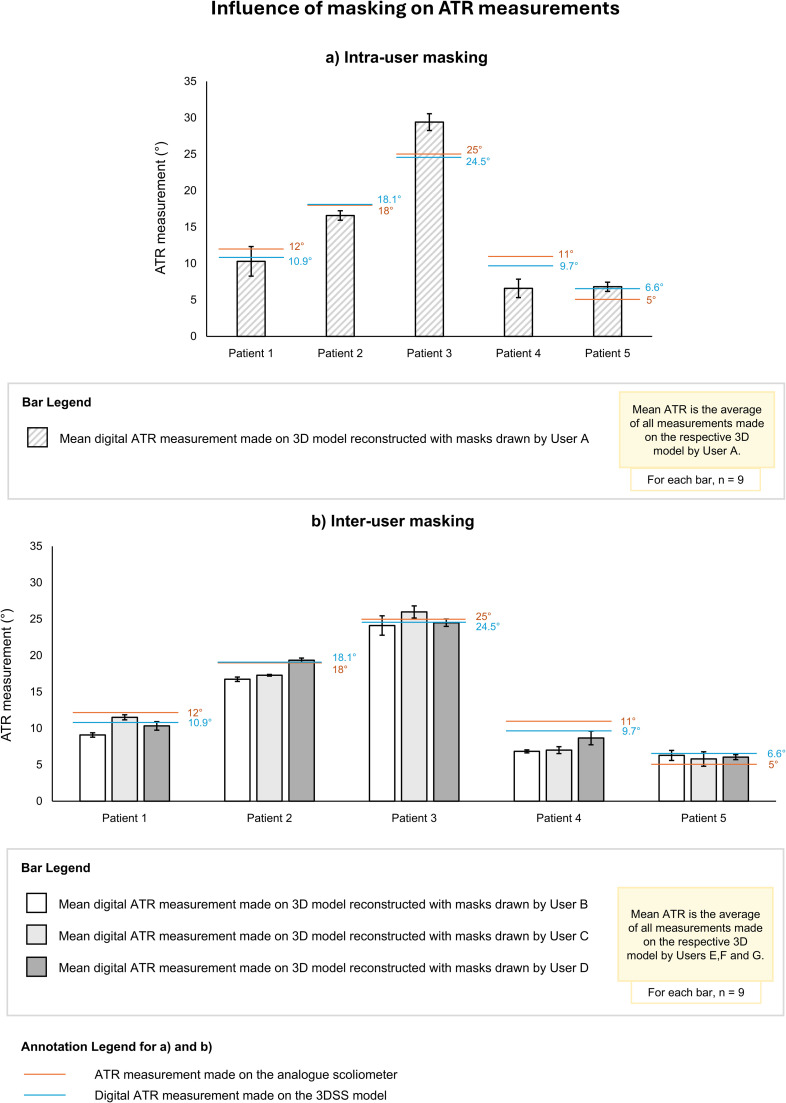
Mean digital ATR measurement for 5 patients masked by (A) one user 3 times (User A) and (B) once by 3 different users, with ATR digital measurement completed by Users E, F, and G.

Average ATR measurements for each of the 5 patients when masked 3 times by User A are shown in Fig. 7. Average ATR measurements for each of the 5 patients when masked by 3 different users (B, C, and D) are also shown in Fig. 7. For both analyses, the difference in measurement to the clinical analog standard and 3DSS standard was consistently ≤5°.

Average measurement error across all ATR measurement attempts by User A across 3 different mask sets was 1.5 ± 1.3°. Average measurement error across all ATR measurement attempts by User E across 3 different mask sets (by User B, C, and D) was 1.5 ± 0.9°. Similarly, for User F it was 1.4 ± 0.8° and for User G it was 1.3 ± 0.8°.

### Influence of scaling method on ATR measurements

**Fig. 8:**
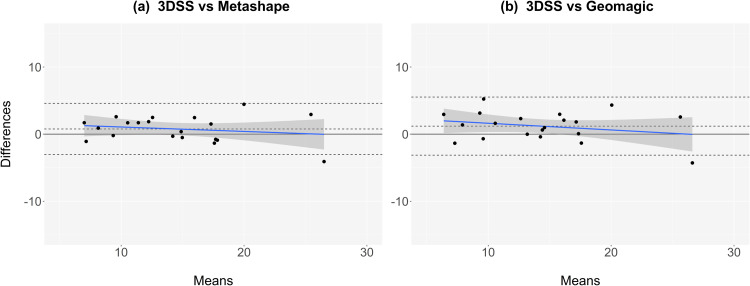
Bland-Altman plots comparing ATR measurements from 3DSS compared with smartphone SfM when scaling was performed on 2 different software - (a) Agisoft Metashape and (b) Geomagic Wrap. The blue line represents the proportional bias, the gray shading represents the 95% confidence interval for the proportional bias line, and the dotted lines represent the limits of agreement. For each plot, *n* = 20.

Comparisons of ATR measurements taken from differently scaled models are graphed in Bland-Altman plots in Fig. 8. A mean bias of 0.78° was observed when comparing ATR measurements made on SfM models scaled in Metashape compared to those made on 3DSS models. A mean bias of 1.13° was observed when comparing ATR measurements made on SfM models scaled in Geomagic compared to those made on 3DSS models.

## Discussion

SfM offers an inexpensive alternative to more advanced measurement technologies, as it can be performed using images captured with standard consumer-grade digital cameras or even smartphones, and the entire workflow can be processed using freely accessible software [34]. This proof-of-concept study shows evidence that single-smartphone SfM is a viable alternative to 3DSS for application in spine deformity when conducted by a trained professional. It can be used to reliably measure clinically relevant angular surface metrics like the ATR, on par with analog clinical measurements and 3DSS measurements.

The methods described in this study have been developed with the intention of testing and implementing at-home smartphone SfM capture in the future. In this study, patients were imaged on a wooden calibration board for consistency with 3DSS protocols developed in the past. For at-home use, any object of known size can be used for size calibration. For example, milk/juice cartons, cereal boxes, small stools/chairs/tables, large boxes of tissues, board game boxes, etc. would all make excellent calibration devices as they have flat sides and sharp edges that can be easily and accurately measured with a ruler. These items are also large enough to be recognized as an independent object in the captured images when the main subject is as large as a human.

The photo capture process described in this paper is simple and easy to follow. To support future nonexpert image acquisition by a parent/guardian, a standardized instruction using a “clock-face” analogy was developed. Preliminary pilot testing of the protocol was conducted in a clinical setting, wherein parents were instructed to capture images of their own children following the described method. These findings informed protocol refinement showing that the number of photos required for the generation of good quality 3D models from which clinical measurements can be made is reasonable for a parent/guardian to capture. A formal evaluation of layperson-led image acquisition in home environments is currently underway and will be reported in subsequent work.

The conditions for at-home SfM are expected to be poor and difficult to standardize, particularly with respect to background, lighting conditions, and movement of other members of the household or family pets. As such, the process of masking becomes a critical factor in determining the quality of scan reconstruction. In the current study, an appropriate masking procedure was utilized for application in spine deformity, which was robust and resilient to moving backgrounds, different lighting conditions, and cluttered spaces.

Clinically, it is accepted that analog measurement of ATR using a scoliometer in the AFB pose will vary by up to 5° to 8° [[35], [36], [37]]. The mean measurement variability for the ATR when masking images from SfM, in combination with the digital scoliometer, is under 2° regardless of the user who completes the masking or the user who makes the ATR measurement. This variability is similar to that seen in 3DSS measurements and is lower than what is seen in the analog scoliometer [19]. We show that this is applicable to a clinic setting, where there may be multiple clinical staff who process 3D models for telehealth appointments, and staffing may not be consistent over a period of time. We also show that masking by a novice with minimal instruction is feasible, and a moderate familiarity with 3D tools is sufficient to produce quality 3D models from which the ATR can be measured. We acknowledge, however, that this assessment was performed with a very small subset of data due to the significant rater burden. Future work on automating this masking process with artificial intelligence tools would be highly beneficial not only in reducing the manual work, but also in reducing the variability in masking contours between users.

Scaling of the 3D models, which was performed on different software, had a negligible impact on ATR measurements. This study, however, did not investigate scaling errors in detail (eg, comparing the scale drift between different methods and different users). Scaling assessments are often conducted in the field of computer vision, typically for large terrain mapping [26,34], and the acceptable error in reconstructed 3D models varies with the capture size and whether the subject is stationary or not [27]. While live human captures are gaining popularity in the medical field, scale errors are scarcely investigated in isolation in the context of the relevant medical application [25,32] and would be highly valuable to do so in future research.

The ability of an experienced operator to reliably capture patient images using a smartphone for SfM reconstruction has been demonstrated in this study. However, further work is required to evaluate whether lay users can effectively follow the proposed protocol in a home environment. This includes both positioning the child correctly in the AFB pose and acquiring structured image sets, including any calibration objects. Beyond image acquisition, successful clinical translation of this approach requires development across other stages of the pipeline [19]. Ongoing work within our research group includes the creation of clear user-centered, culturally responsive educational resources (eg, written instructions, visual guides, and video demonstrations) to support at-home scanning. Mechanisms for secure storage and transfer of sensitive health data are also being established, ensuring compliance with local data privacy and medical regulatory standards. In addition, we are exploring robust methods for quality control and automated validation of uploaded image sets to identify incomplete or suboptimal captures prior to reconstruction. Finally, procedures to integrate the reconstructed 3D models and their associated measurements into existing hospital information systems and clinical workflows are currently being formulated to enable efficient clinician access and longitudinal tracking, thereby supporting routine clinical use.

Smartphones have become nearly ubiquitous, with an estimated 7.3 billion subscriptions worldwide as of 2025 [38]. As such, they have become a useful tool in telehealth infrastructure, helping to enhance health outcomes in rural areas by improving access. It also lessens the financial stress associated with receiving critical tertiary spine care, thereby improving socio-economic wellbeing [39]. In addition, early research on the impact of smartphone usage on health outcomes shows promise in increasing patient engagement in their care trajectory and better facilitating the dissemination and understanding of health information. It has been shown that patients engaged in their care report higher levels of care satisfaction, increased knowledge about diagnosis, testing and treatment, have more realistic expectations, are more likely to adhere to treatment plans and, in some cases, even have improved health outcomes [[40], [41], [42]]. Implementing methods like at-home SfM in a telehealth context is expected to have positive ramifications for narrowing the gap between patient and clinician.

## Conclusions

This study is a first assessment into using smartphone SfM to enhance telehealth assessments of spine deformity, particularly AIS. We investigated the feasibility of suboptimal image capture conditions, such as moving backgrounds, lighting variations, and cluttered spaces, with a view to implementing “at-home scanning” in the future. In addition, we assessed the impact of 2 manual processes within the SfM 3D model reconstruction pipeline, such as manual background masking and scaling. The accuracy of 3D model generation was evaluated in the context of the hospital tertiary outpatient clinic with the measurement of the ATR. This study shows that single-smartphone SfM is a viable low-cost alternative to 3DSS, bringing the implementation of surface topography a step closer to integration within hospital outpatient clinic telehealth systems.

## Declarations of competing interests

The authors declare that they have no known competing financial interests or personal relationships that could have appeared to influence the work reported in this paper.

## Appendix Supplementary materials

Image, application 1
