# Supplementary material for: Smartphone-based Structure-from-Motion for the remote assessment of trunk rotation in spine deformity
Source: N Am Spine Soc J. 2026 May 17;27:100900. doi: 10.1016/j.xnsj.2026.100900 (PMC13277533; doi:10.1016/j.xnsj.2026.100900)
Supplement: Supplementary file 1 [file mmc1.docx]

**
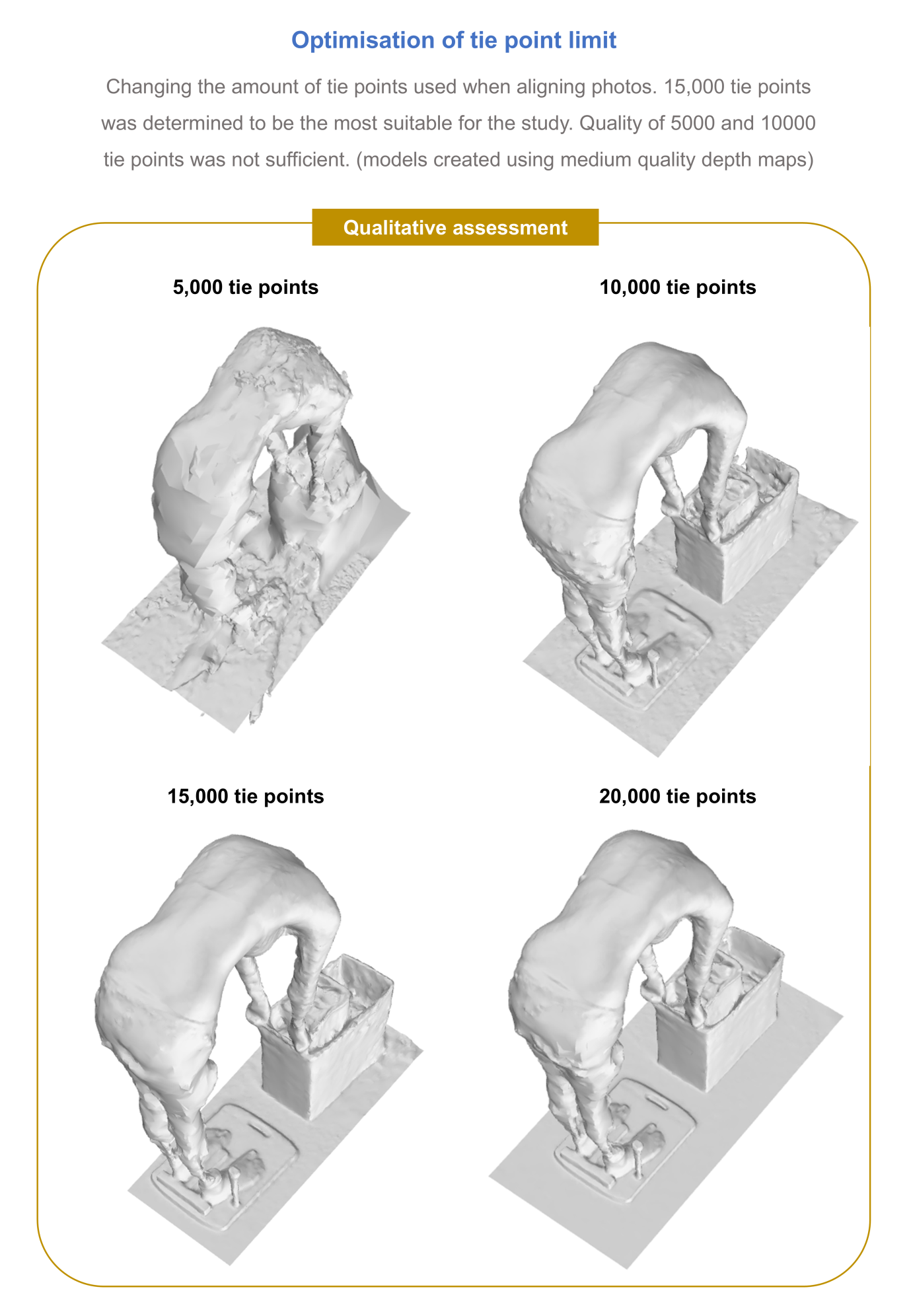
**

**Supplementary Figure 1 –** Qualitative optimisation of tie point limit for reconstruction of SfM models on Agisoft Metashape.

**
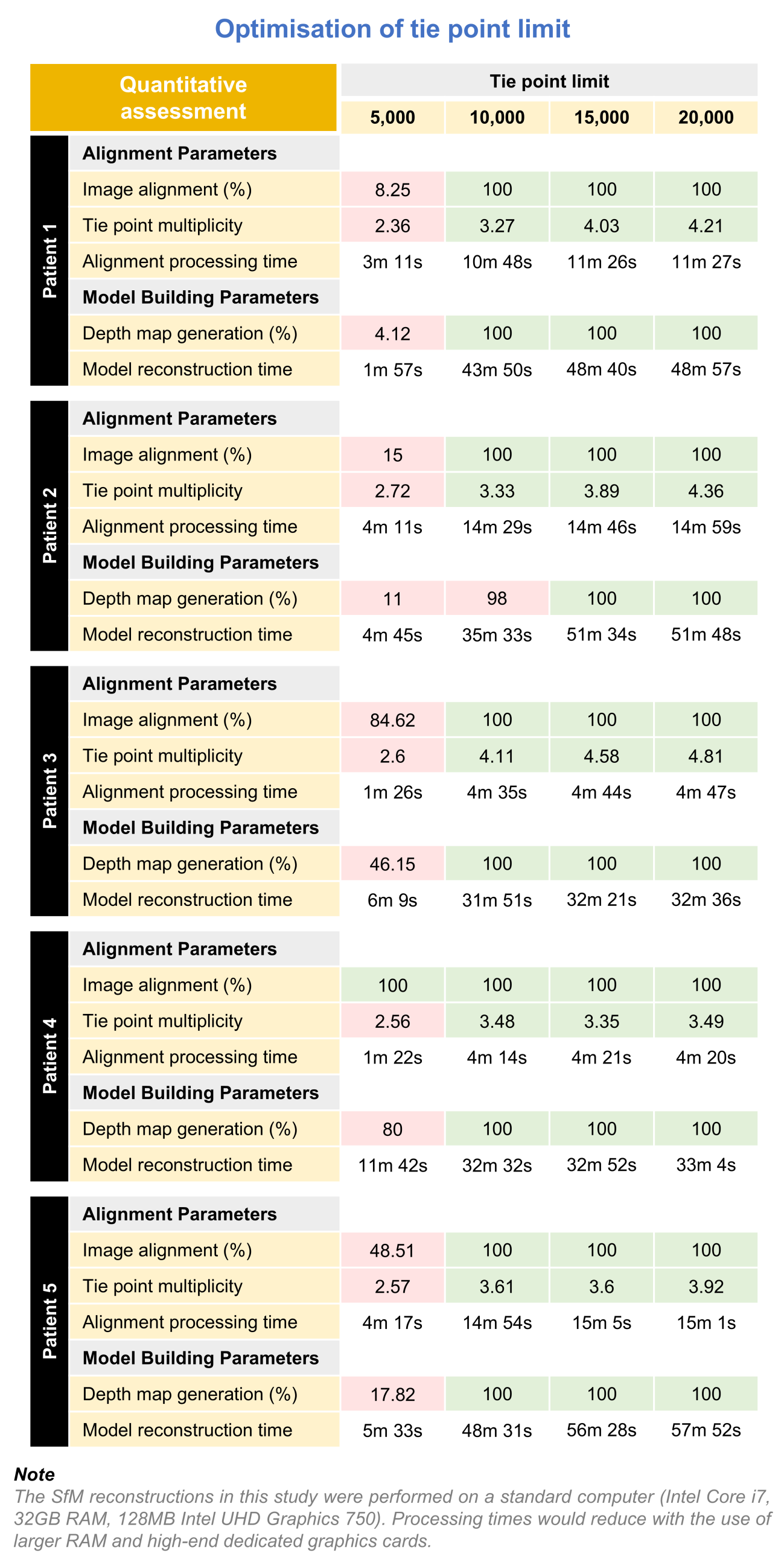
**

**Supplementary Figure 2 –** Quantitative optimisation of tie point limit for reconstruction of SfM models on Agisoft Metashape.

**
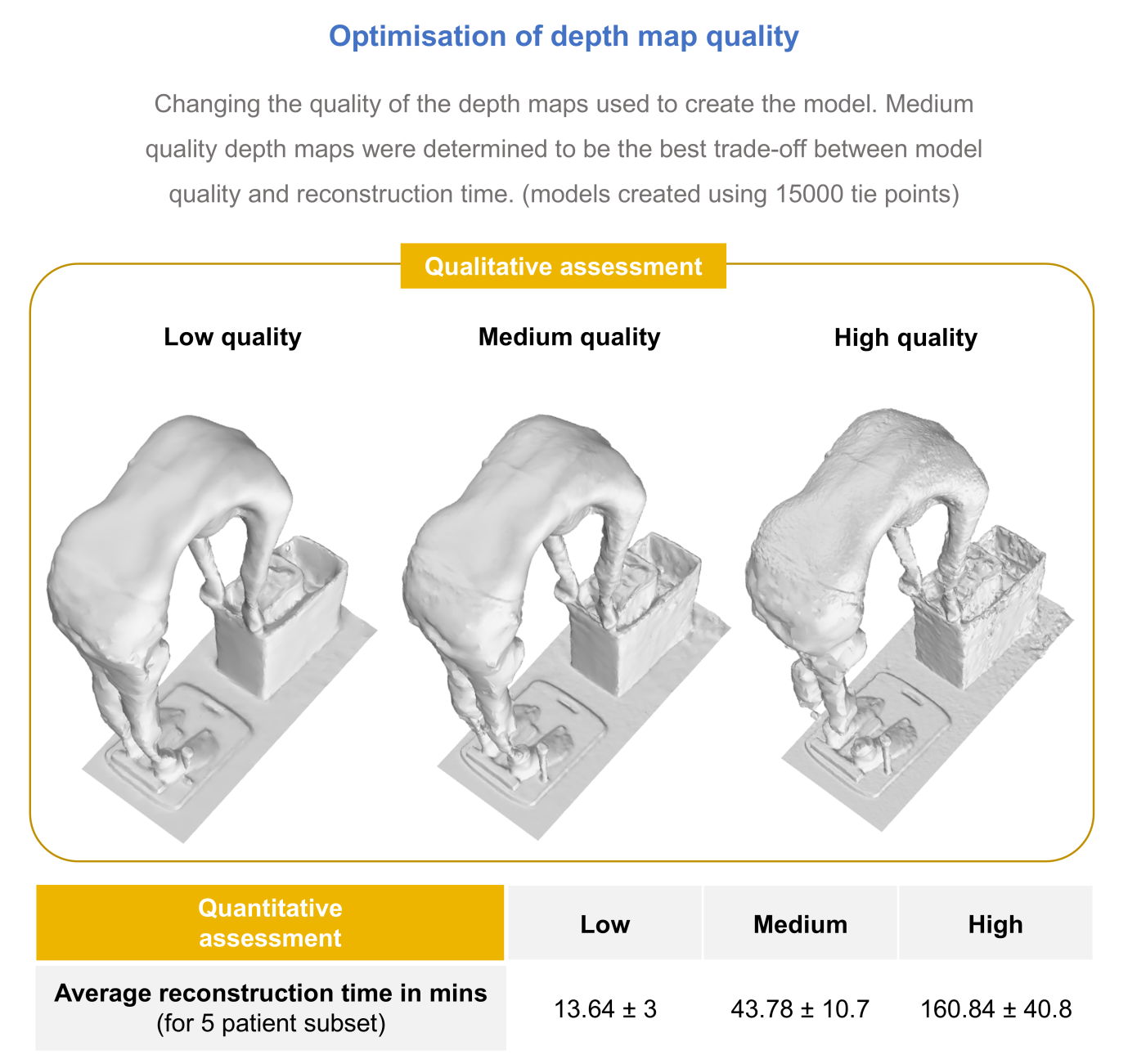
**

**Supplementary Figure 3 –** Qualitative and quantitative optimisation of depth map quality for reconstruction of SfM models on Agisoft Metashape.

**
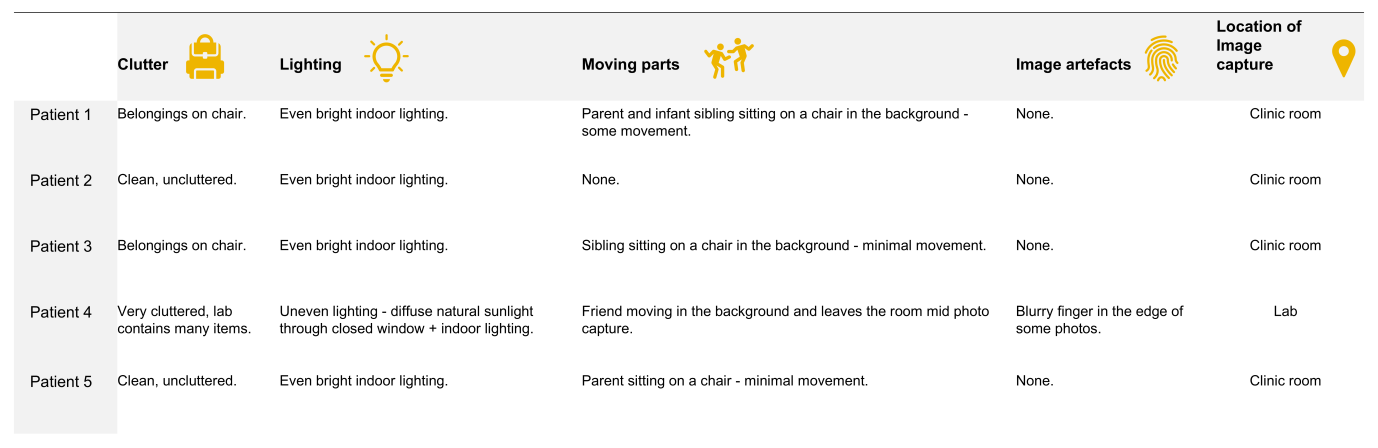
**

**Supplementary Figure 4 –** Background conditions for SfM capture for a subset of five patients, used for the assessment of image masking.
